# A Multi-Omics Analysis Reveals Anti-Osteoporosis Mechanism of Four Components from Crude and Salt-Processed *Achyranthes bidentata* Blume in Ovariectomized Rats

**DOI:** 10.3390/molecules27155012

**Published:** 2022-08-06

**Authors:** Yuwen Yin, Fei Zhu, Meiling Pan, Jiaqi Bao, Qing Liu, Yi Tao

**Affiliations:** 1 Zhejiang Technical Institute of Economics, Hangzhou 310032, China; 2 College of Pharmaceutical Science, Zhejiang University of Technology, Hangzhou 310014, China

**Keywords:** *Achyranthes bidentata*, anti-osteoporosis, network pharmacology, metabolomics, microbiome

## Abstract

The root of *Achyranthes bidentata* Blume (AB) is a well-known traditional Chinese medicine for treating osteoporosis. Plenty of studies focused on the pharmacological mechanism of the whole extract; however, the contribution of different components to the anti-osteoporosis effect remains unknown. The aim of this study is to explore the anti-osteoporosis mechanism of different components of crude and salt-processed AB under the guidance of network pharmacology, metabolomics, and microbiomics. First, network pharmacology analysis was applied to constructing the compound-target-disease network of AB to provide a holistic view. Second, the anti-osteoporosis effects of the four components were evaluated in female Wistar rats. The subjects were divided into a normal group, a model group, a 17α-estradiol (E2)-treated group, a polysaccharide-component-treated groups, and a polysaccharide-knockout-component-treated groups. All the serum, urine, and feces samples of the six groups were collected after 16 weeks of treatment. Biochemical and microcomputed tomography (μCT) parameters were also acquired. Coupled with orthogonal partial least-squares discrimination analysis, one dimensional nuclear magnetic resonance (NMR) was used to monitor serum metabolic alterations. A total of twenty-two biomarkers, including lipids, amino acids, polyunsaturated fatty acids, glucose, and so on were identified for the different components-treated groups. Through pathway analysis, it is indicated that glyoxylate and dicarboxylate metabolism, glycine, serine, and threonine metabolism, alanine, aspartate, and glutamate metabolism, d-glutamine, and d-glutamate metabolism were the major intervened pathways. Levels of these biomarkers shifted away from the model group and were restored to normal after treatment with the four components. In addition, 16S rDNA sequencing demonstrated that the abundance of *Anaerofilum*, *Rothia*, and *Turicibacter* bacteria was positively correlated with an anti-osteoporosis effect, whereas the abundance of *Oscillospira* was negatively correlated. The osteoprotective effect of the polysaccharide components of crude and salt-processed AB is related to the regulation of the abundance of these gut microbiota.

## 1. Introduction

By 2022, the number of senior citizens over 60 years old will increase to about 267 million in China [1]. The aging of the population makes osteoporosis a noteworthy issue since osteoporosis can seriously endanger the health of the senior citizens [2]. Osteoporosis is mainly divided into postmenopausal osteoporosis and senile osteoporosis. Postmenopausal women are the biggest victims of the disease due to the dual effects of aging and the decline of estrogen levels in the body, accounting for more than 70% of the patients [3]. Thus far, the drugs for the prevention and treatment of osteoporosis include anti-resorptive agents, bone enhancers, and mineralization agents, all which bear several adverse effects, such as liver and kidney damage, digestive tract irritation, and cold-like symptoms [4]. With the long history in the treatment of osteoporosis, traditional Chinese medicine owns the advantages of systemic conditioning, less side effects, and low price [5].

*Achyranthes bidentata* Blume (AB), which belongs to *Amaranthaceae,* is an herb widely distributed in the Henan Province of China [6]. The roots of AB have been applied in traditional Chinese medicine for hundreds of years as an anti-osteoporosis agent [7]. After being salt processed, the herb will exert enhanced effects in strengthening the muscles and bones. Several kinds of chemical constituents [6,8], particularly phytosterone, triterpenoids, and polysaccharides have been identified from the roots of AB. Our group applied ultra-performance liquid chromatography/quadrupole time-of-flight mass spectrometry (UPLC-Q-TOF/MS) to determine eight main chemical compounds in different batches of crude and salt-processed AB [9]. Meanwhile, the pharmacokinetic behaviors of five compounds were compared after oral administration of crude and salt-processed AB to rats [10]. Previous pharmacological study showed that the anti-inflammatory, antioxidant, and anti-apoptotic effects of salt-processed AB were much better than that of crude herbs [11]. Despite this, a vast body of research focused on the osteoprotective effect of crude AB extracts [12,13]. However, the comparison of metabolic and gut microbiota profiles between crude and salt-processed AB on osteoporosis rats is still rare.

Metabolomics is an exciting and evolving research field with numerous success stories indicating that its power extends from biomarker discovery to understanding the mechanisms that underlie diseases [14]. For metabolomic analysis, both NMR and MS have their strengths and weaknesses. NMR instruments do not need to be recalibrated nearly as often as MS instruments, and sample preparation of NMR is extremely simple. Meanwhile, NMR has been widely used for evaluating therapeutic effects and toxic effects of Chinese herbal medicine [15,16]. Recent research confirmed that gut microbiota plays a pivotal role in bone metabolism and the pathogenesis of osteoporosis [17,18,19]. In addition, AB polysaccharide can promote growth and improve intestinal morphology and the microbiota community structure of broilers [20].

The aim of this study is to explore the osteoprotective mechanism of polysaccharide components and polysaccharide knockout components from crude and salt-processed AB with a multi-omics analysis method. To explore the osteoprotective effect of four components of crude and salt-processed AB, 16S rRNA-gene-sequencing-based microbiomics were combined with NMR-based metabolomics methods.

## 2. Materials and Methods

### 2.1. Chemicals and Reagents

The roots of AB were collected in Jiaozuo City, Henan Province in November 2020 (batch number: 20201114) and authenticated by Professor Ping Wang. Voucher specimens of the herb was deposited in the Moganshan campus of Zhejiang University of Technology. D_2_O (99.9% D) was purchased from Sigma Co. (Darmstadt, Germany). K_2_HPO_4_ and NaH_2_PO_4_ were obtained from Xilong Chemical Co (Huanggu County, Shenyang, China). Phosphate buffer solution (pH 7.4) was prepared by dissolving 50 mM K_2_HPO_4_/NaH_2_PO_4_ in the D_2_O. Standard substances, including L-valine, L-serine, L-proline, d-mannose, L-alanine, L-cysteine, L-leucine, L-lysine, L-threonine, L-aspartate, L-phenylalanine, and palmitic acid were obtained from Shanghai Ryon Biological Technology Co (Qingpu County, Shanghai, China). Citrate, succinic acid, glycine, glucose, cholesterol, and stearate and were purchased from Sinopharm Chemical Reagent Co (Jingan County, Shanghai, China).

### 2.2. Preparation of Four Components of Crude and Salt-Processed AB

The salt-processing procedure was carried out according to the 0213 general rules of processing in the 2020 edition of Chinese Pharmacopeia. Briefly, a hundred grams of crude roots of AB was rinsed with brine (∼2 g salt) for 2 h. After that, the crude root was transferred to a preheated wok. Gentle heat was used to stir-fry the crude roots. The temperature of the stir-frying was about 165 °C. The whole procedure was stopped until the root was dried. As a result, the salt-processed AB was allowed to cool at room temperature until further experiments.

The extraction procedures for crude and salt-processed AB were according to previous reports with slight modifications [11]. First, a hundred grams of crude or salt-processed AB were extracted with water (×8, *V*/*V*) at 100 °C two times, and the extracts were concentrated in vacuo. Second, the obtained concentrated extract was placed in a beaker, and ethanol was slowly added while stirring until the alcohol percentage of the solution reached 80%. After standing for 24 h, the supernatant was removed and concentrated to be the polysaccharide knockout component, whereas the sediment at the bottom was freeze-dried to be the polysaccharide component. The polysaccharide knockout components of crude and salt-processed AB were analyzed using UPLC-Q-TOF/MS. The polysaccharide components of crude and salt-processed AB were analyzed using gel permeation chromatography. The representative total ion chromatogram and gel permeation chromatogram are provided in Figures S1 and S2, Supplementary Materials.

### 2.3. Experimental Design

**Figure 1:**
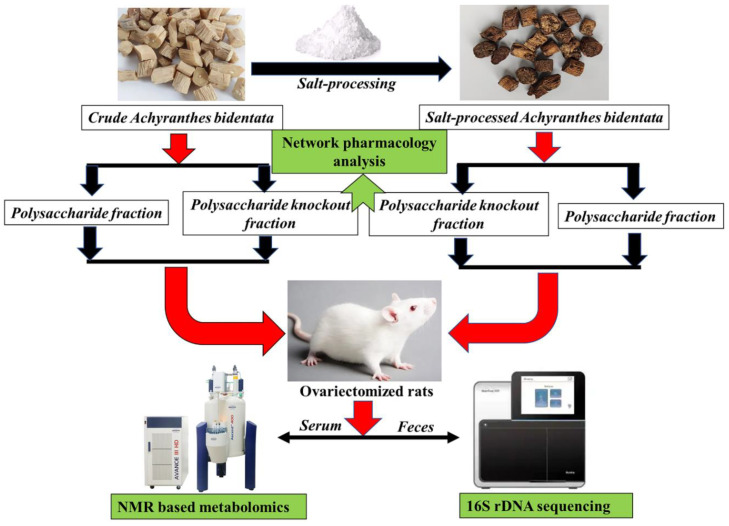
The schematic flowchart of the combinatory strategy for dissecting the anti-osteoporosis mechanism of four components from crude and salt-processed AB.

The experimental design of this study is displayed in Figure 1. The sample size for each group is four. Animal experiments are the topics which have been discussed for a long time. Minimizing the use of laboratory animals is not just a requirement of the IACUC (Institutional Animal Care and Use Committee) but also of the animal welfare protector. Animal experiments have many shortcomings, especially differences in species leading to the difficulty of extrapolation of experimental data. It is now stressed that through strict design, only the “necessary” number of animals could be used. Biochemical and biomechanical indexes were also acquired to confirm the success of the osteoporosis model. The serum metabolic profile of different components-treated groups were analyzed by using the NMR-based metabolomic method. Meanwhile, multivariate statistical analysis was used to identify potential biomarkers and related metabolic pathways. Gut bacterial variation was analyzed by using 16S rDNA sequencing and correlated with bone mineral intensity. These observations will lead to a better understanding of the relationship between bone homeostasis and the microbiota in osteoporosis and shed light on the osteoprotective effect of different components of crude and salt-processed AB.

### 2.4. Network Pharmacology Analysis

The chemical composition of AB was obtained by searching the database of PubChem and PubMed. Then, ChemDraw18.0 software was used to draw compound structures and export the Smiles file of each compound. The targets of each compound were retrieved via the Swiss Target Prediction database and the Similarity Ensemble Approach database. The names of the targets were standardized using the UNIPROT database. The GeneCards database is a human gene database, while the DisGeNET database is a comprehensive gene-disease-associated-relationship database. “Osteoporosis” is set as the keyword. Osteoporosis-related targets are obtained from two databases. If scores of the targets are above the median value, these targets will be selected. Their names were standardized by browsing the UNIPROT database. Venny 2.1 software was used to pick out common targets of the compounds and osteoporosis, which were introduced into the String database and Cytoscape 3.8.0. A visualized PPI network diagram was constructed with the species setting to “*Homo sapiens*” and confidence > 0.9. In addition, Cytoscape 3.8.0 software was applied to building a “compounds-targets-pathways-disease” network diagram. The plug-in Network Analyzer was used for topological analysis, node freedom, and close-to-centrality calculation. The genes with a score of more than two times the average value were selected as the key targets. The analysis of gene clusters and core target was accomplished through the plug-in, Mcode. To clarify the anti-osteoporosis mechanism of AB, the common genes of AB and osteoporosis are introduced into the David 6.8 database. The parameters of KEGG pathway enrichment analysis and GO biological process enrichment analysis were set as species is “*Homo sapiens*” and threshold *p* < 0.05.

### 2.5. Animals and Drug Administration

All animal experiments were performed under the NIH Guidelines for Care and Use of Laboratory Animals (U.S.A.) and the Prevention of Cruelty to Animals Act (1986) of China. The experiment was approved by the Animal Ethics Committee of Zhejiang University of Technology (No.20210916). All female Wistar rats (*n* = 24), weighing 250 ± 20 g, were obtained from Slaccas Experiment Animal Company (Shanghai, China), and adapted at 22 ± 2 °C, humidified 55 ± 5%, and 12 h light-dark cycle for seven days. The rats were divided into seven groups: normal group; model group (Model); 17*β*-estradiol-treated group (E2); polysaccaride component of crude-AB-treated group (ABP); polysaccharide knockout component of crude-AB-treated group (AB); polysaccharide component of salt-processed AB-treated group (sABP), and polysaccaride knockout component of salt-processed AB-treated group (sAB). The ovariectomize operation procedure was carried out as described in the previous report [21]. The rats of the E2-treated group received 17*β*-estradiol 25 μg/kg b.w./day dissolved in a mixture of 5% benzyl alcohol and 95% corn oil orally. The rats of the treated groups were orally administered with different extracts of crude and salt-processed AB, respectively. According to the 2020 edition of Chinese Pharmacopeia, the routine dose of AB for human was 5–12 g/day. Therefore, rats received an equivalent dosage of 1.08 g/kg/day. All the samples were suspended in distilled water. Rats in the normal and model groups received the same volume of distilled water. After treatment for 16 weeks, the rats were transferred to metabolic cages and subjected to a 12 h fast to collect urine sample. Abdominal aorta blood was collected from the rats.

### 2.6. Micro-CT and Dual-Energy X-ray Absorptiometry Analysis

The right femora which had been stored at −80 °C were thawed prior to bone scanning. The femora were scanned by small animal micro-CT (ZKKS, Guangzhou, China) at a pixel size of 18 µm. The 3D images were acquired for visualization. Bone mineral intensity (BMD), trabecular bone volume (BV/TV), trabecular thickness (Tb.Th), trabecular number (Tb.N), and trabecular separation/spacing (Tb.Sp) were obtained. Moreover, the right femora were sent for dual-energy X-ray absorptiometry measurements. A quality control scan was undertaken at the start and end of each scanning session. The scanning was carried out according to the literature [21]. The maximum load, stiffness, energy, maximum stress, and young modulus were recorded.

### 2.7. Biochemical Index Determination

The serum was sent to be analyzed using a Hitachi 7100 automated biochemical analyzer. Concentrations of calcium (Ca), phosphorus (P), and creatinine (Cr) of urine and the alkaline phosphatase (ALP) activity of the serum were obtained.

### 2.8. ^1^H-NMR Spectroscoy Based Metabolic Profiling

The serums were thawed in ice, and 350 μL aliquots were mixed with 350 μL PBS (pH 7.4) to minimize variations in pH. Then, all samples were centrifuged at 12,000× g for 10 min at 4 °C and transferred into a 5 mm NMR tube. All ^1^H-NMR spectra were acquired at 298 K on the Bruker AVANCE III at 600 MHz. One-dimensional spectra were acquired by using the Carr–Purcell–Meiboom–Gill (CPMG) pulse sequence (RD-90°-(τ-180°-τ)_n_-ACQ) with water suppression. The total spin–spin relaxation delay was set to 80 ms to attenuate broad NMR signals of macromolecules and retain signals of metabolites. The spectral width was 20 ppm with an acquisition time of 1.64 s, and a total of 256 free induction decays were collected into 64 k data points for each spectrum.

The NMR spectra were manually phased, corrected for baseline correction, referenced to the lactate (CH_3_, at δ1.33 ppm), and carefully aligned using MestReNova (version 9.0). The spectral region of δ0.0–9.0 ppm was segmented into 9000 bins with a width of 0.001 ppm. The residual integrals from the region of δ4.6–5.1 ppm in suppressed water resonance were excluded in all spectra. Each sample data was normalized to the sum of the spectral intensity to compensate for differences in the concentrations of samples.

### 2.9. 16S rDNA Sequencing

The feces samples of the six groups were sequenced individually using an Illumina Miseq platform. DNA extraction, PCR amplification, sequencing, and data analysis were performed successively. Briefly, gut microbial genomic DNA was extracted by using a TIANamp Stool DNA Kit. Then, the V3–V4 region of the 16S rRNA gene was amplified with the well-established universal primers, 338F (5′-ACTCCTACGGGAGGCAGCA-3′) and 806R (5′-GGACTACHVGGGTWTCT AAT-3′).The amplification of the gene was carried out as follows: initial denaturation for 3 min at 95 °C, 30 cycles each of denaturation for 30 s at 95 °C, annealing for 30 s at 55 °C, and primer extension for 45 s at 72 °C. The amplicons were purified using the Ampure XP beads and quantified with a Qubit 3.0 fluorometer using a Qubit dsDNA HS Assay Kit.

### 2.10. Multivariate Statistical Analysis

All statistical analyses of regular biochemical indices were carried out by using the Minitab 14.0 statistical package (Minitab, Inc., Pennsylvania, USA). Statistical differences among the groups were assessed by one-way ANOVA. Values were considered statistically significant at *p* < 0.05. The normalized data of NMR were imported into the SIMCA-P software (Version 13.0). The principal component analysis (PCA) model approximates the variation in a data table by a low-dimensional model plane. Then, the partial least squares discriminant analysis (PLS-DA) and orthogonal signal correction partial least squares discriminant analysis (OPLS-DA) were used to classify the samples. S-plot and VIP plots were used to extract the correlated variables in relevance with the sample belongings. Pearson correlation was performed between bone mineral intensity and the abundance of gut microbiota.

## 3. Results and Discussion

### 3.1. Network Pharmacology Analysis

**Figure 2:**
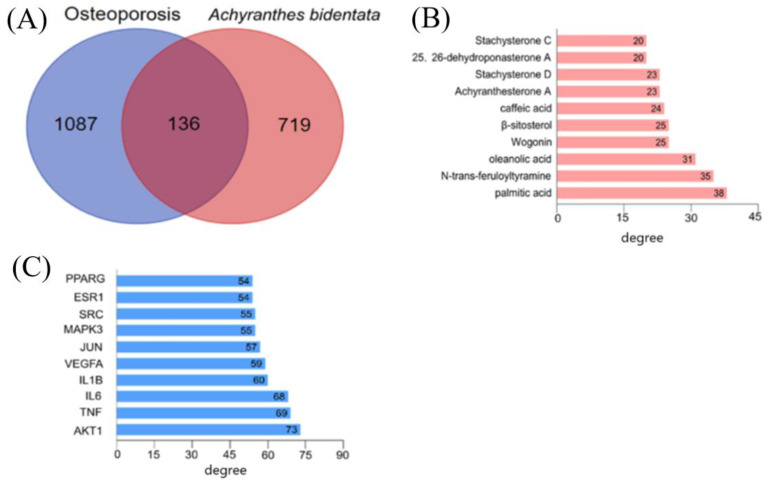
Network pharmacology analysis of crude and salt-processed AB on treating osteoporosis: (**A**) Venn plot, (**B**) top 10 compounds, (**C**) top 10 targets.

A total of one hundred and thirty-three chemical constituents of AB were collected from the literature and database, including forty-nine triterpenoids, ten ketosteroids, seven sterols, five alkaloids, six flavonoids, three anthraquinones, twelve organic acids, and thirty-four other types. Through the screening of the Swiss Target Prediction database and SEA database, 855 targets of chemical constituents of AB were obtained. Using the OMIM, GeneCards, and DisGENET databases, 1223 OP-related genes were obtained. A total of 136 targets were shared in Venn plot (see Figure 2A), which were imported into the STRING platform to construct a PPI network diagram (see Figure S3, Supplementary Materials). The biological species was set to “*Homo sapiens*”, and the target with the lowest interaction threshold greater than 0.9 was selected to construct the PPI network diagram. The PPI network graph has a total of 136 nodes and 1246 edges with an average degree of 18.3.

The ingredient-target-disease network diagram was drawn using the software, Cytoscape 3.8.0 (see Figure S4, Supplementary Materials). The purple diamond represents the compound. Blue circles are the osteoporosis targets. Using the Network Analyzer to perform topology analysis on the network graph, the top 10 compounds of node degree were obtained, as shown in Figure 2B. The abscissa is the degree value of each compound. Compounds with high node degree are the key compounds of AB, which play an important role in the treatment of osteoporosis. The top 10 compounds are palmitic acid, N-trans-feruloyltyramine, oleanolic acid, wogonin, β-sitosterol, caffeic acid, achyranthesterone A, stachysterone D, 25,26-dehydroponasterone A and stachysterone C. The genes with scores greater than two times the average score were selected as key targets. A total of 32 key targets were screened, and the first 10 targets are listed in Figure 2C. The abscissa is the degree value of each target. The therapeutic effect of AB on osteoporosis is mainly related to protein kinase B (AKT1), tumor necrosis factor (TNF), interleukin 6 (IL-6), interleukin 1B (IL1B), vascular endothelial growth factor A (VEGFA), JUN, mitogen-activated protein kinase 3 (MAPK3), SRC, estrogen receptor 1 (ESR1), and peroxisome proliferative activated receptor gamma (PPARG).

### 3.2. Biochemical Indexes

Several biochemical indexes were determined. Compared with the normal group, the level of S-Ca was significantly decreased in the model group. The levels of S-Ca and S-P among other groups showed no significant different (see Table 1), which is in agreement with that of the literature [22]. The ratios of U-Ca/Cr and U-P/Cr levels in the E2-treated group were significantly lower than that of the model group. Treatment with the four components of crude and salt-processed AB could significantly reduce the U-Ca/Cr level in OVX rats. Notably, treatment with the polysaccharide component of salt-processed AB showed the largest changing amplitude. ALP is an important indicator for osteoporosis. The four components treatment could attenuate the activity of ALP in the serum of OVX rats.

### 3.3. Micro-CT and Dual-Energy X-ray Absorptiometry Analysis

**Figure 3:**
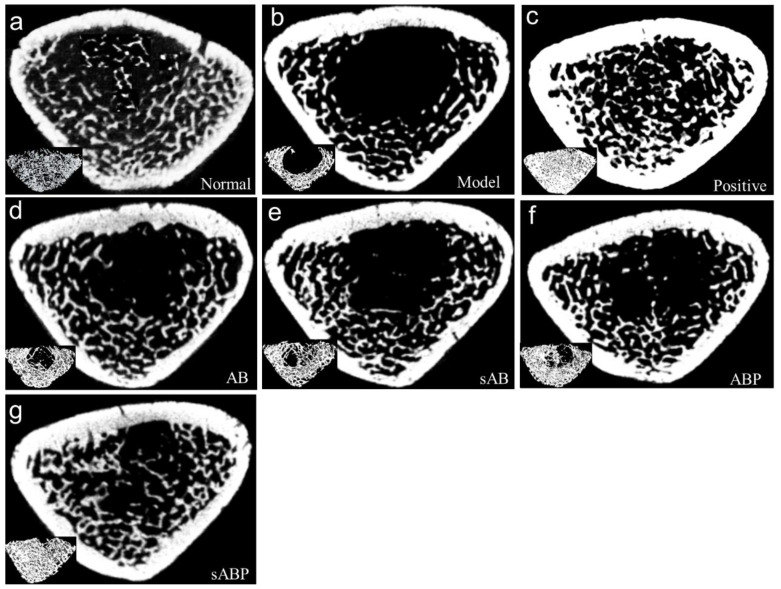
Representative 2D and 3D mapping architectures of trabecula bone of different groups: (**a**) normal group, (**b**) model group, (**c**) positive group, (**d**) polysaccharide knockout component of crude AB-treated group, (**e**) polysaccharide knockout component of salt-processed AB-treated group, (**f**) polysaccharide component of crude AB-treated group, (**g**) polysaccharide component of salt-processed AB-treated group.

Compared with the normal group, the BMD, BV/TV, Tb.Th, and Tb.N levels of the model group were significantly decreased. Compared with the model group, E2 treatment significantly increased the right femur bone mineral density (BMD) (see Table 2). Meanwhile, the four components of crude and salt-processed AB treatment significantly increased the right femur BMD compared to the model group. As shown in Figure 3a, the analysis of 2D and 3D mapping architectures of the trabecular bone clearly showed that ovariectomy resulted in the deterioration of the trabecular bone microarchitecture in rats, which was also validated by the reduction of BV/TV, Tb.Th, and Tb.N levels (see Table 2). Treating OVX rats with E2 or the four components of crude and salt-processed AB could significantly reverse the alterations in these indexes induced by ovariectomy and could restore the microarchitecture of the trabecular bone in the proximal metaphysis of the rats’ femora (see Figure 3b–f).

Table 3 shows the bone biomechanical indexes of the femoral diaphysis of rats in different groups, including maximum load, stiffness, energy, maximum stress, and young modulus. Compared with the normal group, the five bone biomechanical indexes of the model group were significantly decreased. Treatment with E2 and the four components of crude and salt-processed AB significantly elevated the five biomechanical indexes. Collectively, the osteoprotective effect of the polysaccharide component of salt-processed AB seems to be better than that of the other three components but does not exceed the osteoprotective effect of E2.

### 3.4. Metabolic Profile Analysis of Four Components from Crude and Salt-Processed AB

**Figure 4:**
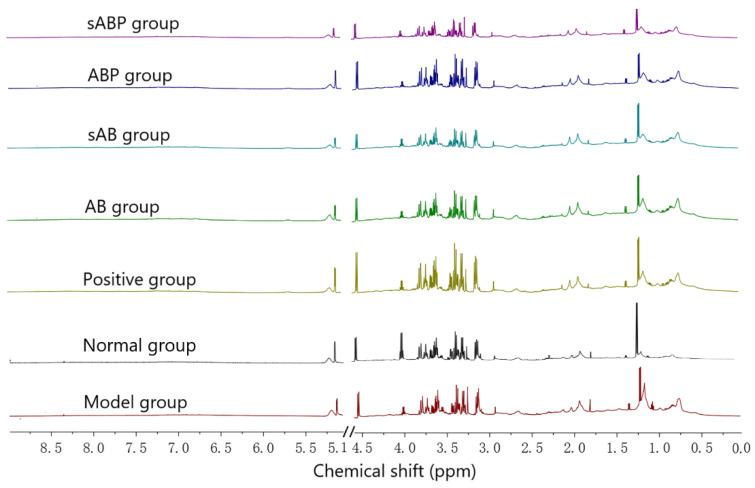
Representative NMR spectra of serum samples from normal group, model group, and differently treated groups.

Representative NMR spectra of serum samples from different groups are displayed in Figure 4.

**Figure 5:**
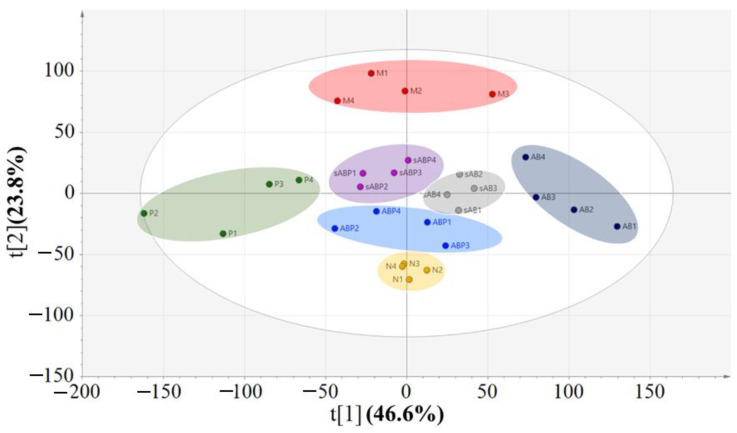
Score plot of PCA model of normal group, model group, and differently treated groups. N1-N4 represents normal group, M1–M4 represents model group, P1–P4 represents E2-treated group, AB1–AB4 represents polysaccharide knockout component of crude AB-treated group, sAB1–sAB4 represents polysaccharide knockout component of salt-processed AB-treated group, ABP1–ABP4 represents polysaccharide component of crude AB-treated group, sABP1–sABP4 represents polysaccharide component of salt-processed AB-treated group.

**Figure 6:**
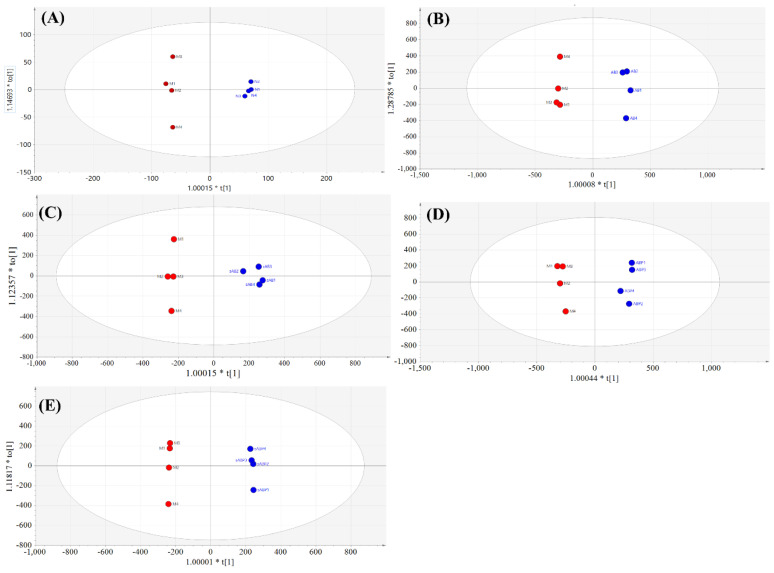
Score plots of OPLS-DA models: (**A**) model group vs. normal group, (**B**) model group vs. polysaccharide knockout component of crude AB, (**C**) model group vs. polysaccharide knockout component of salt-processed AB, (**D**) model group vs. polysaccharide component of crude AB, (**E**) model group vs. polysaccharide component of salt-processed AB.

**Figure 7:**
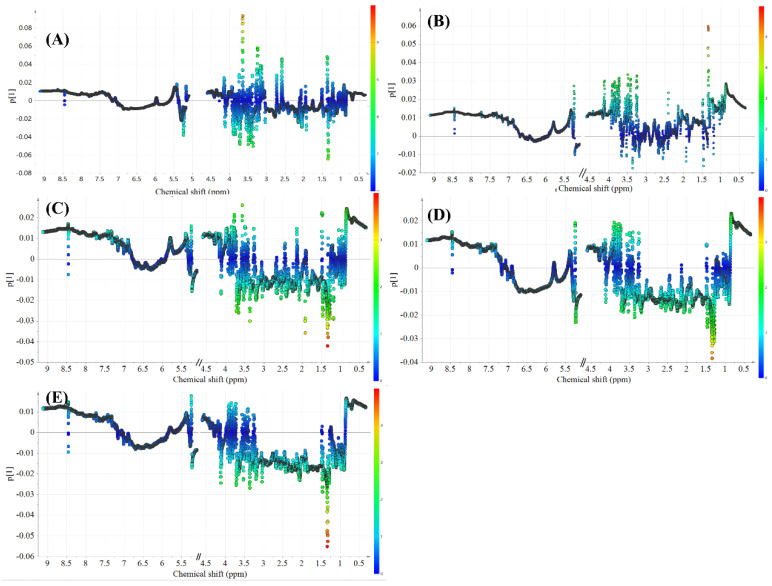
Loading plots of OPLS-DA models: (**A**) model group vs. normal group, (**B**) model group vs. polysaccharide knockout component of crude AB, (**C**) model group vs. polysaccharide knockout component of salt-processed AB, (**D**) model group vs. polysaccharide component of crude AB, (**E**) model group vs. polysaccharide component of salt-processed AB.

Distinct separation trends among the normal group, model group, E2-treated group, and four components from the crude and salt-processed AB-treated groups were observed in the PCA score plot (see Figure 5). Subsequently, orthogonal partial least squares-discrimination analyses (OPLS-DAs) were applied to classifying the model group and the four components-treated groups. The score plots and loading plots of the model group versus the polysaccharide knockout component of crude AB, model group versus polysaccharide knockout component of salt-processed AB, model group versus polysaccharide component of crude AB and model group versus polysaccharide component of salt-processed AB are displayed in Figure 6A–D and Figure 7A–D.

**Figure 8:**
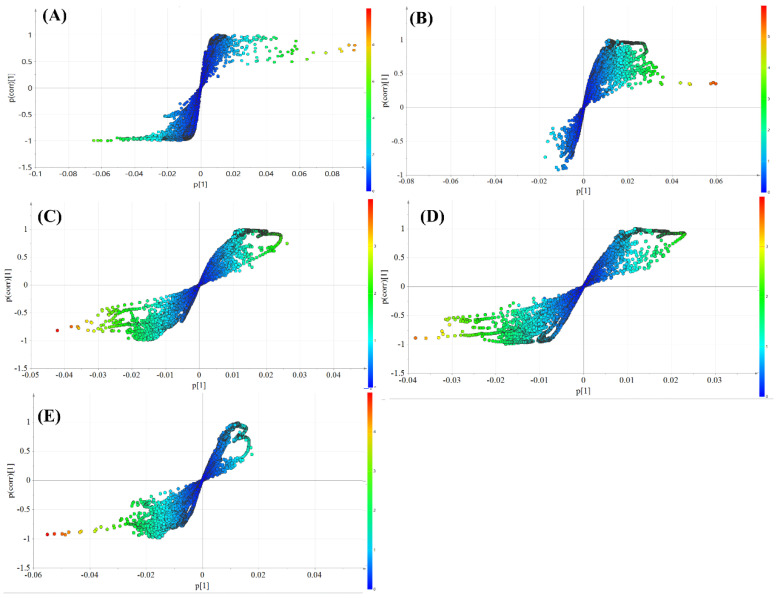
S-plot of different groups: (**A**) model group vs. normal group, (**B**) model group vs. polysaccharide knockout component of crude AB, (**C**) model group vs. polysaccharide knockout component of salt-processed AB, (**D**) model group vs polysaccharide component of crude AB, (**E**) model group vs. polysaccharide component of salt-processed AB.

The S-plots were generated to identify the significant varied endogenous metabolites with a variable importance in the projection (VIP) of more than 1.0 in the NMR data, and the results are shown in Figure 8A–D. To analyze these differential metabolites globally, the varied endogenous metabolites are listed in Table 4. Compared with the normal group, 18 differential metabolites (VIP > 1.0) were observed in the model group. Compared with the model group, 13 and 19 differential metabolites (VIP > 1.0) were observed in the polysaccharide knockout component of crude and salt-processed AB-treated group, respectively. Meanwhile, 21 and 22 differential metabolites (VIP > 1.0) were identified when comparing metabolites between the polysaccharide component of crude and salt-processed AB treated group and model group.

**Figure 9:**
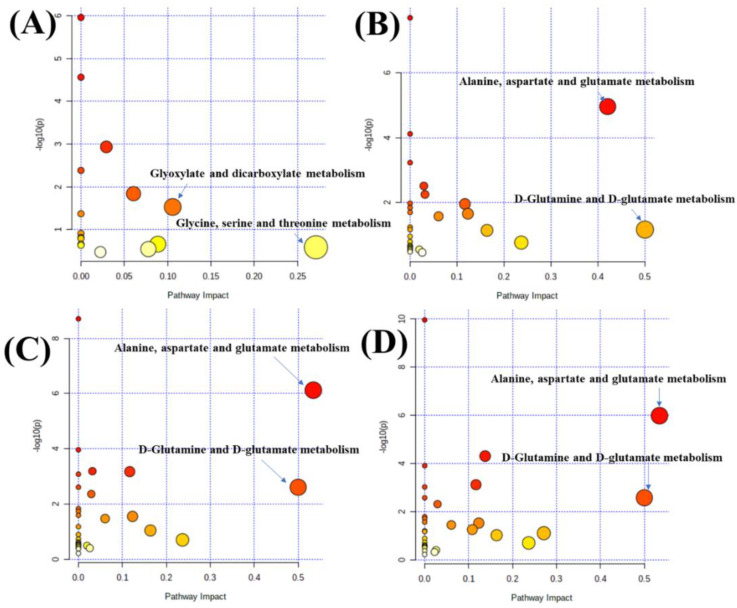
Overview of pathways related to the differential endogenous metabolites in different treated groups: (**A**) polysaccharide knockout component of crude AB-treated group, (**B**) polysaccharide knockout component of salt-processed AB-treated group, (**C**) polysaccharide component of crude AB-treated group, (**D**) polysaccharide component of salt-processed AB-treated group.

To undermine the anti-osteoporosis mechanism, MetaboAnalyst 3.0 was applied to the metabolic pathway analysis. Pathway impact plots were generated to visualize the impact of altered metabolic pathways (see Figure 9). The detailed illustration of Figure 9 is shown in Figures S3–S6, Supplementary Materials. Treatment with polysaccharide knockout component of crude AB mainly regulated the pathways involving glyoxylate and dicarboxylate metabolism and glycine, serine, and threonine metabolism. Intriguingly, treatment with the other three components intervened alanine, aspartate, and glutamate metabolism and d-glutamine and d-glutamate metabolism.

Glycolysis is an important part of carbon flux in cell proliferation [23]. Lactate is the terminal product of glycolysis. Citrate and succinate are intermediate products of the citrate cycle. Compared with the model group, the levels of lactate, citrate, and succinate in the sAB-, ABP-, and sABP-treated groups were decreased. This phenomenon can be explained by the bone resorption of osteoclasts consuming a large amount of energy, which causes the speed up of glycolysis and oxidative phosphorylation [24]. With the enhancement of energy metabolism, the tricarboxylic acid cycle (TCA) is also enhanced. As an important intermediate product of the TCA, the concentrations of citrate and succinate in serum is largely increased. This finding is consistent with that of previous studies [25]. The decrease of the lactate, citrate, and succinate levels in the treated groups demonstrated that the glycolysis and TCA cycle of OVX rats was shifted to the normal state after treatment. In the model group, a large amount of glucose was consumed, and the glucose concentration in serum became lower. To compensate for the large consumption of glucose, glycogenic amino acids (i.e., serine, glycine, alanine) tend to participate into the glycolysis process to produce more glucose [22]. For instance, the decreased levels of glycine and serine in the model group implied that the activated glycine and serine entering the glycolysis pathway was largely consumed. Our finding agrees with the report that osteoporosis led to increased glycine and serine activation [26]. The elevation of glycine and serine levels in the AB- and sAB-treated group indicated the restoration of the TCA cycle and energy metabolism.

Glutamine regulates bone metabolism through osteoclasts and can be converted into glutamate, which may cause bone resorption through the expression of glutamate receptors on bone cells [27]. It is reported that elevated glutamine and low content of BMD are highly associated [28]. Our study found the levels of glutamine, glutamate, and aspartate were significantly increased in the model group. After treatment with the four components of crude and salt-processed AB, the levels of glutamine, glutamate, and aspartate decreased dramatically which revealed the shift of these three amino acids in OVX rates to the direction of normal state.

3-Hydroxybutyrate is an end product of fatty acid β-oxidation. The high level of 3-hydroxybutyrate and low level of lipid may suggest that osteoporosis enhanced fatty acid β-oxidation to support the energy demand. The activated fatty acid β-oxidation has been confirmed in previous research [28]. The decrease in the level of 3-hydroxybutyrate and the increase in the level of lipid were observed in the four components-treated group, indicating the fatty acid β-oxidation was reduced in OVX rats after treatment.

### 3.5. Gut Microbiota Composition of Different Components-Treated Groups

**Figure 10:**
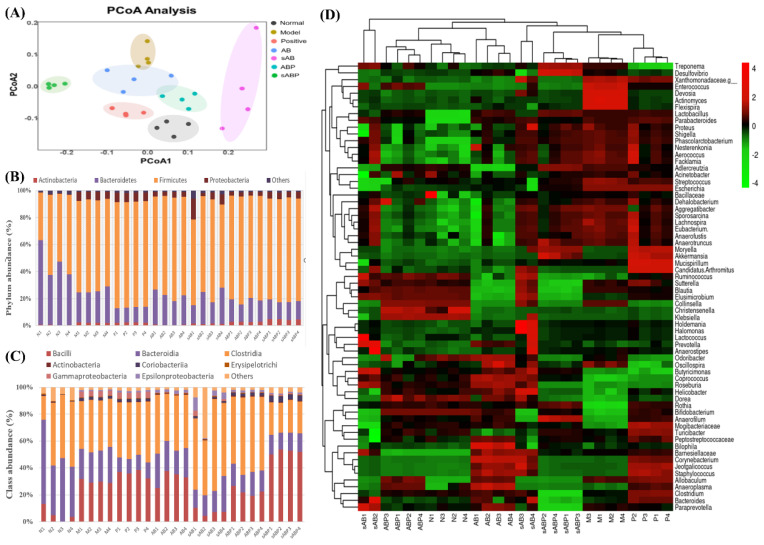
PCoA plot (**A**), phylum abundance (**B**), class abundance (**C**), and general heatmap (**D**) of gut microbiota in feces of normal group, model group, and differently treated groups.

The effects of the four components from crude and salt-processed AB on the gut microbiota composition in OVX rats were investigated by the 16S rRNA sequencing. As is displayed in Figure 10A, the principal coordinates analysis showed the gut microbiota composition of each group was separated from each other. At the phylum level, the relative abundance of *Bacteroidetes* was decreased, whereas the relative abundance of *Furmicutes* was increased in the model group. Our finding is consistent with the literature [29,30]. Treatment with the four components of AB dramatically altered the gut microbiota composition in OVX rats (see Figure 10B). Treatment with AB and sAB increased the relative abundance of *Bacteroidetes* and decreased the relative abundance of *Furmicutes.*

At the class level, the relative abundance of *Bacilli* was significantly increased, while the relative abundance of *Bacteroidia* was decreased in the model group. The oral administration of the four components mainly regulated the relative abundance of *Bacilli* (see Figure 10C). For example, sAB and ABP could decrease the abundance of *Bacilli* compared with the model group. Conversely, oral administration with sABP increase the abundance of *Bacilli* and decrease the abundance of *clostridia*. At the genus level, a heatmap was generated to visualize the abundance of microbiota in the differently treated groups (see Figure 10D).

Pearson correlation analysis was carried out to explore the relationship between the bone mineral intensity level and the gut microbiota abundance. The gut bacterial genera showing moderate to high correlations with BMD are listed in Table 5. The BMD was found to exhibit a high positive correlation with the relative abundance of *Anaerofilum* (r = 0.754), *Rothia* (r = 0.743), and *Turicibacter* (r = 0.729) and a high negative correlation with the relative abundance of *Oscillospira* (r = −0.705). From a multi-omics perspective, *Anaerofilum* was found to be significantly enriched in the model group compared to the normal group. It was discovered that *Anaerofilum* can produce the main metabolite, such as hydrogen sulfide. Accumulated evidence has suggested that hydrogen sulfide (H_2_S) plays a significant role in bone formation and bone tissue regeneration. Interestingly, it was reported that the serum levels of H_2_S were unexpectedly increased in patients with osteoporosis [31]. In contrast, the abundance of *Rothia* and *Turicibacter* were significantly reduced in the model group compared to the normal group. *Rothia* can transform several carbohydrates, including xylose, galactose, raffinose, and glucose, to yield butyric acid. *Turicibacter* is involved in fermentation metabolism to yield lactic acid, which is highly correlated with energy metabolism. The reduction of *Rothia* and *Turicibacter* is associated with gastrointestinal disorders and impaired energy metabolism of osteoporosis rats. Salt-processing is a routine method of Chinese medicine processing. After being salt processed, the effects of strengthening the muscles and bones of AB will be enhanced. Treatment with ABP and sABP restored the abundance of *Anaerofilum, Rothia*, and *Turicibacter* to normal, which may contribute to the osteoprotective effect of crude and salt-processed AB.

## 4. Conclusions

Our study prepared and characterized four components of crude and salt-processed AB, which could significantly prevent ovariectomized-induced osteoporosis and regulate the gut microbiota composition. The sABP component could significantly prevent ovariectomized-induced bone loss, maintain metabolism homeostasis, and alleviate dysbiosis of the gut microbiome, suggesting the priority of sABP to be developed for treating osteoporosis. Moreover, the bone mineral intensity level was found to be associated with the relative abundance of *Anaerofilum*, *Rothia*, *Turicibacter,* and *Oscillospira*. However, the detailed interaction of the microbes with endogenous metabolites needs further investigation. Our findings highlight the novel application of polysaccharides of salt-processed AB in the prevention of osteoporosis is related to the regulation of gut microbiota.

## Figures and Tables

**Table 1:** Biochemical parameters of serum and urine of rats in different groups.

Parameters	Model Group	E2-Treated Group	AB-Treated Group	sAB-Treated Group	ABP-Treated Group	sABP-Treated Group
S-Ca (mM)	2.50 ± 0.07	2.47 ± 0.03	2.52 ± 0.05	2.51 ± 0.05	2.54 ± 0.04	2.47 ± 0.04
S-P (mM)	1.58 ± 0.07	1.64 ± 0.11	1.60 ± 0.09	1.64 ± 0.07	1.62 ± 0.10	1.67 ± 0.11
U-Ca/Cr (mmol/mmol)	0.37 ± 0.01	0.20 ± 0.02 **	0.30 ± 0.02 **	0.29 ± 0.01 **	0.27 ± 0.01 **	0.25 ± 0.01 **
U-P/Cr (mmol/mmol)	4.76 ± 0.11	3.74 ± 0.17 **	4.41 ± 0.15 **	4.49 ± 0.09 **	4.20 ± 0.25 **	4.13 ± 0.24 **
ALP (U/L)	268.90 ± 9.11	129.92 ± 7.19 **	217.67 ± 14.65 **	210.36 ± 14.29 **	181.78 ± 11.47 **	173.48 ± 16.67 **

Values are mean ± S.E.M. ** *p* < 0.05 vs. model group as evaluated by ANOVA.

**Table 2:** μCT 3-D parameters of trabecula bone in the distal femur region.

Parameters	Model	E2	AB	sAB	ABP	sABP
BMD (g/cm^3^)	0.348 ± 0.011	0.768 ± 0.039 **	0.459 ± 0.013 **	0.401 ± 0.012 **	0.490 ± 0.014 **	0.543 ± 0.016 **
BV/TV	8.292 ± 0.331	43.345 ± 0.527 **	16.405 ± 0.857 **	13.174 ± 0.818 **	17.903 ± 0.587 **	23.402 ± 0.560 **
Tb.Th (mm)	0.079 ± 0.005	0.102 ± 0.004 **	0.080 ± 0.006	0.079 ± 0.002	0.080 ± 0.007	0.077 ± 0.004
TB.N (1/mm)	1.051 ± 0.050	4.178 ± 0.063 **	2.024 ± 0.039 **	1.590 ± 0.041 **	2.098 ± 0.061 **	2.799 ± 0.055 **
Tb.Sp (mm)	1.033 ± 0.029	0.137 ± 0.008 **	0.451 ± 0.054 **	0.501 ± 0.037 **	0.436 ± 0.013 **	0.250 ± 0.036 **

Values are mean ± S.E.M. ** *p* < 0.05 vs. model as evaluated by ANOVA.

**Table 3:** Bone biomechanical indexes of the femoral diaphysis of rats in different groups.

Parameters	Model Group	E2-Treated Group	AB-Treated Group	sAB-Treated Group	ABP-Treated Group	sABP-Treated Group
Maximum load (N)	103.12 ± 1.94	120.32 ± 1.68 **	109.61 ± 3.77 **	108.47 ± 0.90 **	112.86 ± 1.24 **	115.20 ± 3.84 **
Stiffness (N/mm)	144.97 ± 3.93	159.55 ± 3.87 **	151.80 ± 1.72 **	153.03 ± 4.15 **	155.06 ± 0.90 **	156.09 ± 1.51 **
Energy (N × mm)	39.78 ± 0.59	54.93 ± 3.29 **	44.63 ± 1.96 **	42.37 ± 1.40 **	46.59 ± 2.27 **	50.42 ± 1.28 **
Maximum stress (MPa)	147.88 ± 1.29	202.37 ± 3.70 **	153.51 ± 2.91 **	152.22 ± 4.10 **	159.60 ± 4.67 **	165.60 ± 6.35 **
Young modulus (MPa)	4450.53 ± 52.09	6580.19 ± 102.41 **	5675.56 ± 88.10 **	5153.65 ± 103.23 **	5795.70 ± 63.07 **	5914.54 ± 69.61 **

Values are mean ± S.E.M. ** *p* < 0.05 vs. model group as evaluated by ANOVA.

**Table 4:** Changes in relative levels of metabolites in the serum samples from different groups.

Metabolites	δ(^1^H)	AB-Treated Group vs. Model Group		sAB-Treated Group vs. Model Group		ABP-Treated Group vs. Model Group		sABP-Treated Group vs. Model Group	
		VIP	Change/Fold Change	VIP	Change/Fold Change	VIP	Change/Fold Change	VIP	Change/Fold Change
Lipid	0.83–0.89 (bra)	2.58	↑*/1.33	2.23	↑*/1.27	2.11	↑*/1.33	1.51	↑*/1.16
Leucine	0.96 (t), 1.70 (m), 3.73 (m)	1.27	↓*/1.13	1.29	↑*/1.12	1.20	↓*/0.88	1.33	↓*/0.88
Isoleucine	1.02 (d), 3.73 (m)	1.23	↓*/0.87	1.09	↓*/0.89	1.02	↓*/0.87	1.40	↓*/0.86
Valine	1.05 (d), 3.61 (d)	1.39	↑*/1.21	1.33	↓*/0.77	1.42	↓*/0.67	1.52	↓*/0.73
3-Hydroxybutyrate	1.20 (d), 2.28 (q),2.40 (q), 4.15 (m)	1.05	↑*/1.30	1.62	↓*/0.78	1.34	↓*/0.77	1.56	↓*/0.79
Lactate	1.33 (d), 4.12 (q)	5.52	↑*/2.04	3.94	↓*/0.87	1.12	↓*/0.82	4.98	↓*/0.79
Alanine	1.48 (d), 3.78 (q)	1.62	↑*/1.36	2.03	↑*/1.31	1.34	↑*/1.21	1.41	↓*/0.84
Acetate	1.92 (s)	1.29	↓*/0.83	2.69	↓*/0.72	1.67	↓*/0.80	1.67	↓*/0.81
Glutamate	2.08 (m), 2.34 (m)	—	—	1.22	↓*/0.89	1.48	↓*/0.82	1.89	↓*/0.81
Glutamine	2.13 (m), 2.45 (m)	—	—	—	—	1.22	↓*/0.72	1.61	↓*/0.84
Succinate	2.37 (s)	—	—	1.48	↓*/0.81	1.45	↓*/0.73	1.71	↓*/0.77
Citrate	2.54 (d), 2.66 (d)	—	—	1.74	↓*/0.66	1.66	↓*/0.56	1.37	↓*/0.70
Aspartate	2.87 (m), 2.94 (m)	—	—	1.21	↓*/0.77	1.29	↓*/0.64	1.35	↓*/0.72
Choline	3.20 (s)	—	—	1.82	↓*/0.78	1.60	↓*/0.72	1.47	↓*/0.78
Proline	3.36 (m)	1.61	↓*/0.80	1.70	↓*/0.83	1.82	↓*/0.73	2.18	↓*/0.75
Glycine	3.57 (s)	1.03	↑*/1.27	—	—	—	—	1.04	↓*/0.83
Glucose	3.24 (q), 3.48 (t), 3.90 (q), 3.54 (t), 3.71 (t), 3.83 (t)	3.05	↑*/1.45	1.18	↓*/0.89	1.36	↑*/1.15	1.65	↓*/0.81
Serine	3.84 (m), 3.96 (m)	2.47	↑*/1.46	1.43	↑*/1.17	1.55	↑*/1.22	1.21	↑*/1.14
NAc	2.03 (s)	1.25	↑*/1.13	—	—	1.34	↓*/0.88	1.74	↓*/0.87
PUFA	2.76–2.83 (bra)	—	—	1.17	↓*/0.78	1.23	↓*/0.76	1.02	↓*/0.86
Glycerol	3.61 (m), 3.65 (m)	—	—	2.32	↓*/0.70	1.23	↓*/0.73	1.48	↓*/0.72
Lysine	1.45 (m), 1.71 (m), 1.89 (m), 3.02 (t), 3.75 (t)	—	—	2.51	↓*/0.64	2.20	↑*/1.33	2.62	↓*/0.62

s: single; d: doublet; dd: doublet of doublet; t: triplet; q: quartet; m: multiplet; bra: broad peak; ↑: increase; ↓: decrease; —: no significant change. * *p* < 0 05.

**Table 5:** Pearson correlation coefficients between gut microbiota and anti-osteoporosis effect.

Microbiota	r
*Anaerofilum*	0.754 **
*Bifidobacterium*	0.556 **
*Blautia*	−0.413 **
*Devosia*	−0.469 **
*Lactobacillus*	0.465 **
*Mogibacteriaceae*	0.680 **
*Odoribacter*	−0.565 **
*Oscillospira*	−0.705 **
*Paraprevotella*	−0.428 **
*Peptostreptococcaceae*	0.557 **
*Rothia*	0.743 **
*Turicibacter*	0.729 **

** indicates *p* < 0.05.

## Supplementary Materials

The following supporting information can be downloaded at: https://www.mdpi.com/article/10.3390/molecules27155012/s1.
