# Supplementary material for: A Multi-Omics Analysis Reveals Anti-Osteoporosis Mechanism of Four Components from Crude and Salt-Processed Achyranthes bidentata Blume in Ovariectomized Rats
Source: Molecules. 2022 Aug 6;27(15):5012. doi: 10.3390/molecules27155012 (PMC9370352; doi:10.3390/molecules27155012)
Supplement: Supplementary file 1 [file molecules-27-05012-s001.zip › molecules-1752261-supplementary.pdf]

molecules

MDPI

Supplementary Materials

A Multi-Omics Analysis Reveals Anti-Osteoporosis Mechanism of Four Components from Crude and Salt-Processed *Achyranthes bidentata* Blume in Ovariectomized Rats

Yuwen Yin [1], Fei Zhu [2], Meiling Pan [2], Jiaqi Bao [2], Qing Liu [2] and Yi Tao [2,*]

[1] Zhejiang Technical Institute of Economics, Hangzhou 310032, China.
[2] College of Pharmaceutical Science, Zhejiang University of Technology, Hangzhou, 310014, China
* Correspondence: taoyi1985@zjut.edu.cn; Tel./Fax: 86-0571-88320984

Citation: Yin, Y.; Zhu, F.; Pan, M.; Bao, J.; Liu, Q.; Tao, Y. A multi-omics analysis reveals anti-osteoporosis mechanism of four components from crude and salt-processed *Achyranthes bidentata* Blume in ovariectomized rats. *Molecules* **2022**, *27*, 5012. https://doi.org/10.3390/molecules27155012

Academic Editor: Angelo Antonio D'Archivio

Received: 17 May 2022
Accepted: 3 August 2022
Published: 6 August 2022

Publisher's Note: MDPI stays neutral with regard to jurisdictional claims in published maps and institutional affiliations.

Copyright: © 2022 by the authors. Licensee MDPI, Basel, Switzerland. This article is an open access article distributed under the terms and conditions of the Creative Commons Attribution (CC BY) license (https://creativecommons.org/licenses/by/4.0/).

Abstract: The root of *Achyranthes bidentata* blume (AB) is a well-known traditional Chinese medicine for treating osteoporosis. Plenty of studies focused on pharmacological mechanism of whole extract, however, the contribution of different components to the anti-osteoporosis effect remains unknown. The aim of this study is to explore the anti-osteoporosis mechanism of different components of crude and salt-processed AB under the guidance of network pharmacology, metabolomics and microbiomics. First, for the sake of providing a holistic view, network pharmacology analysis was applied to constructing the compound-target-disease network of AB in order to provide a holistic view. Second, anti-osteoporosis effect of the four components was evaluated in female Wistar rats. The subjects were divided into normal group, model group, 17α-estradiol (E2) treated group, polysaccharide component treated groups and polysaccharide knockout component treated groups. All serum, urine and feces samples of the six groups were collected after 16 weeks' treatment. Biochemical and micro computed tomography (μCT) parameters were also acquired. Coupled with orthogonal partial least-squares discrimination analysis, one dimensional nuclear magnetic resonance (NMR) was used to monitor serum metabolic alterations. A total of twenty-two biomarkers, including lipids, amino acids, polyunsaturated fatty acid, glucose and so on, were identified for different components treated groups. Through pathway analysis,it is indicated that glyoxylate and dicarboxylate metabolism, glycine, serine and threonine metabolism, alanine, aspartate and glutamate metabolism, D-glutamine and D-glutamate metabolism were the major intervened pathways. Levels of these biomarkers shifted away from the model group and restored to normal after treatment with the four components. In addition, 16S rDNA sequencing demonstrated that the abundance of *Anaerofilum*, *Rothia*, and *Turicibacter* bacteria was positively correlated with anti-osteoporosis effect, whereas with which the abundance of *Oscillospira* was negatively correlated. The osteoproctective effect of polysaccharide components of crude and salt-processed AB is related to the regulation of the abundance of these gut microbiota.

Keywords: *Achyranthes bidentata*; Anti-osteoporosis; Network pharmacology; Metabolomics; Microbiome

Supplementary Figures

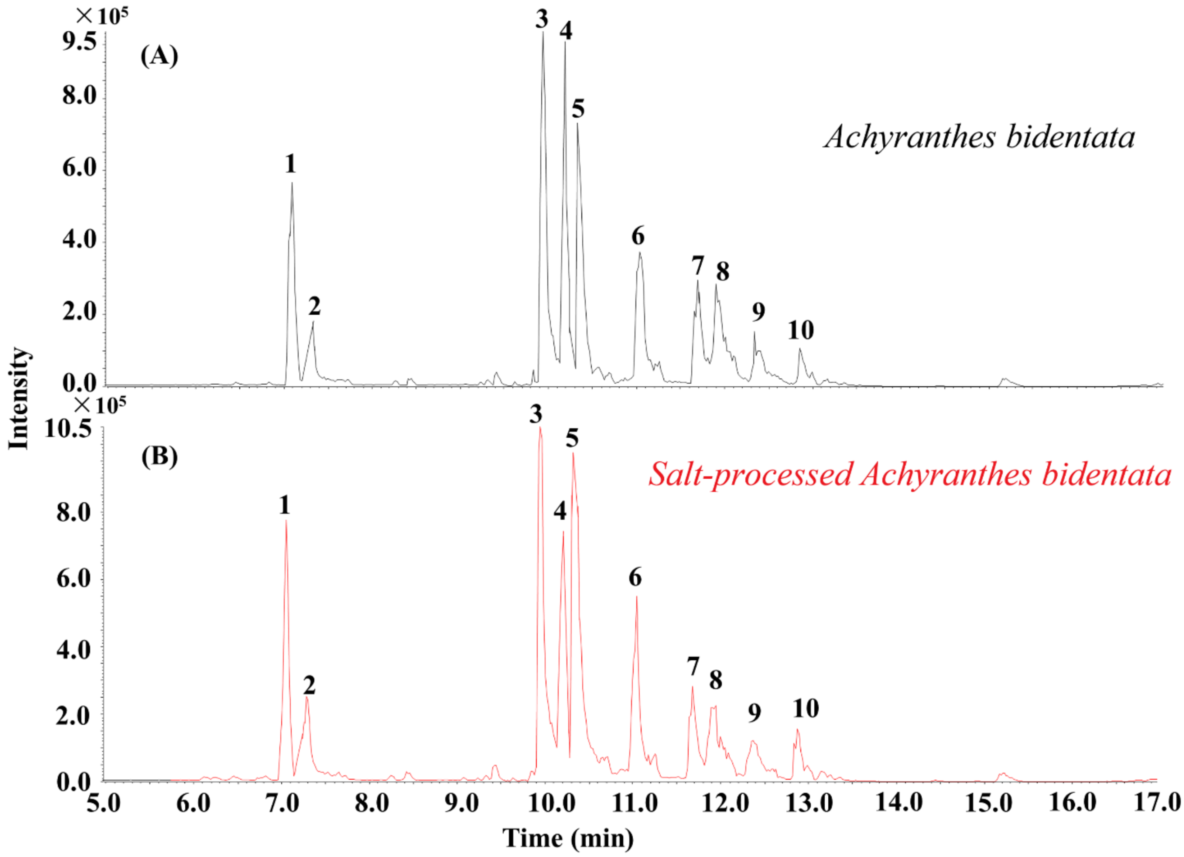

Figure S1. TIC chromatograms of polysaccride knockout components of crude and sal-processed *Achyranthes bidentata*. 1.*β*-ecdysterone, 2. 25*S*-Inokosterone, 3. Achyranthoside D, 4. Ginsenoside Ro, 5. Chikusetsusaponin IV, 6. Achyranthoside C, 7. Chikusetsaponin IVa, 8. 13-HODE.

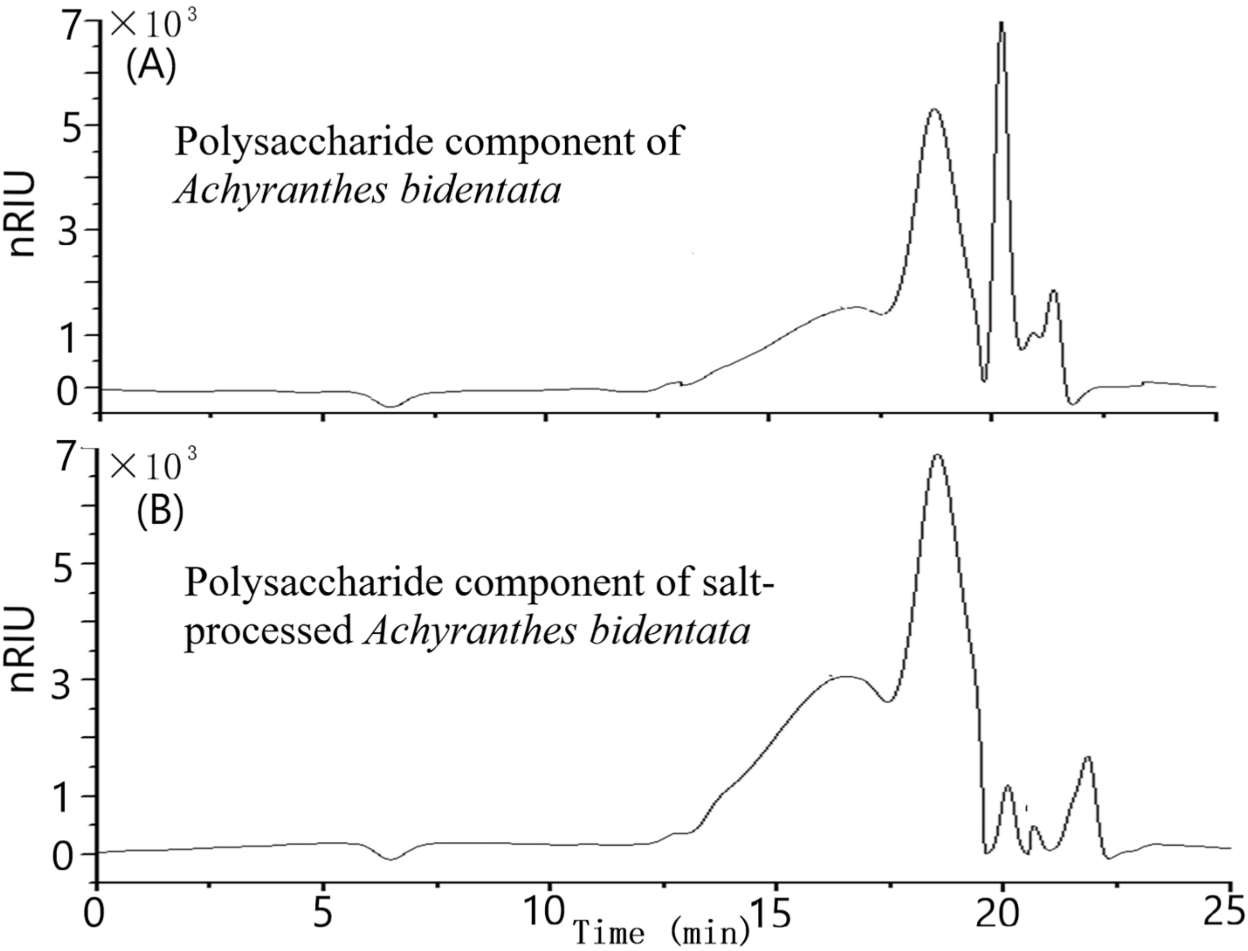

Figure S2. GPC chromatogram of polysaccharide component of crude and salt-processed *Achyranthes bidentata*.

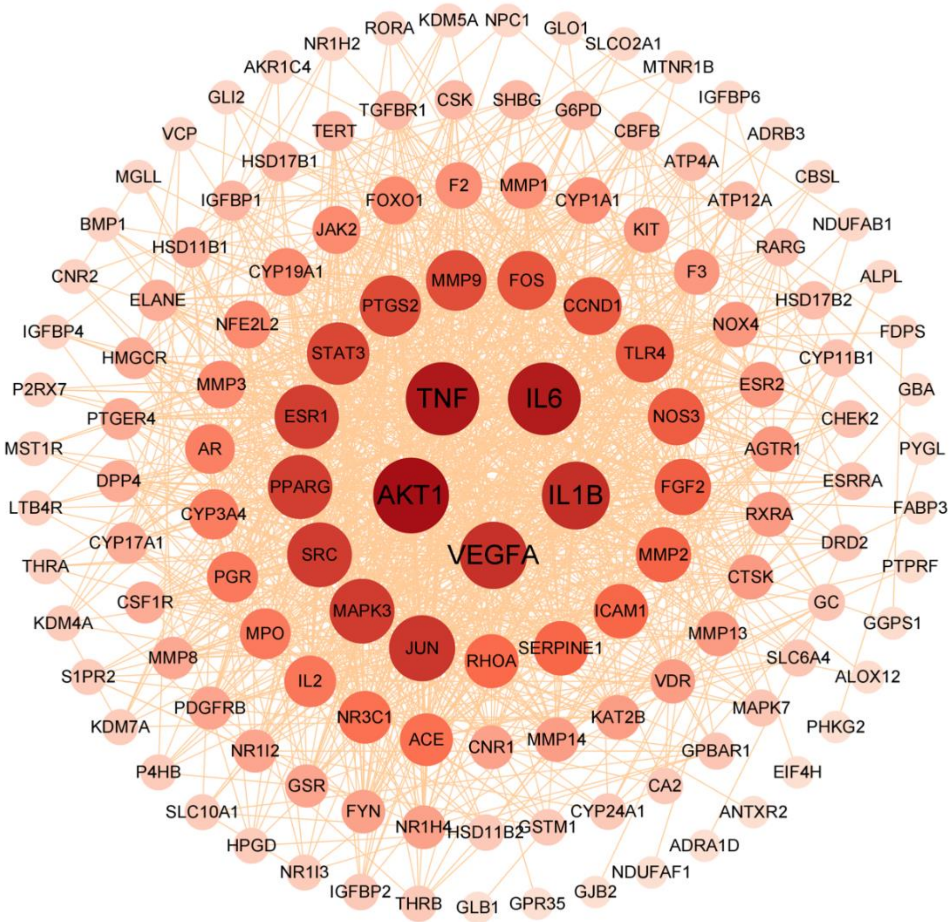

Figure S3. Protein-protein interaction network.

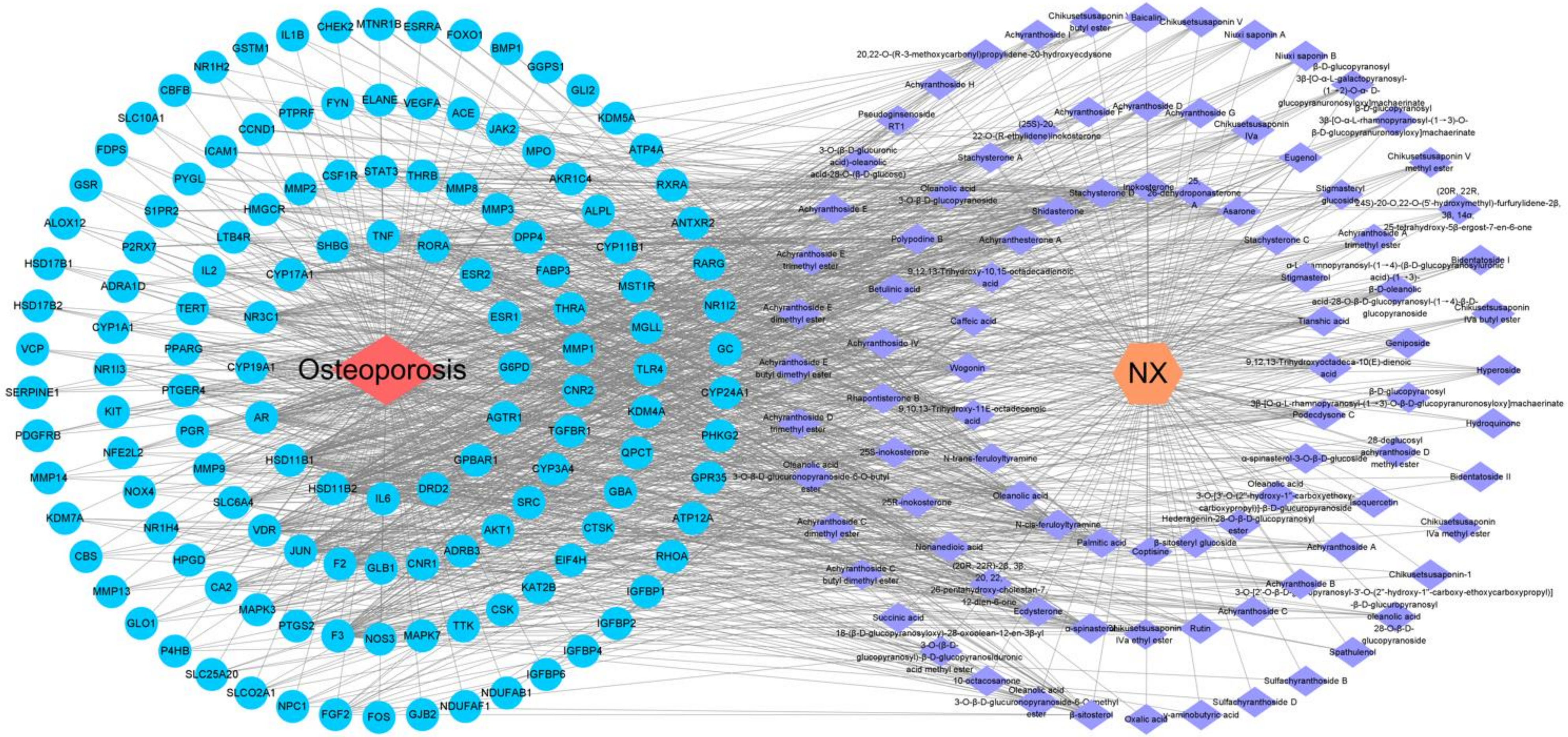

Figure S4. Ingredient-target-disease network diagram.

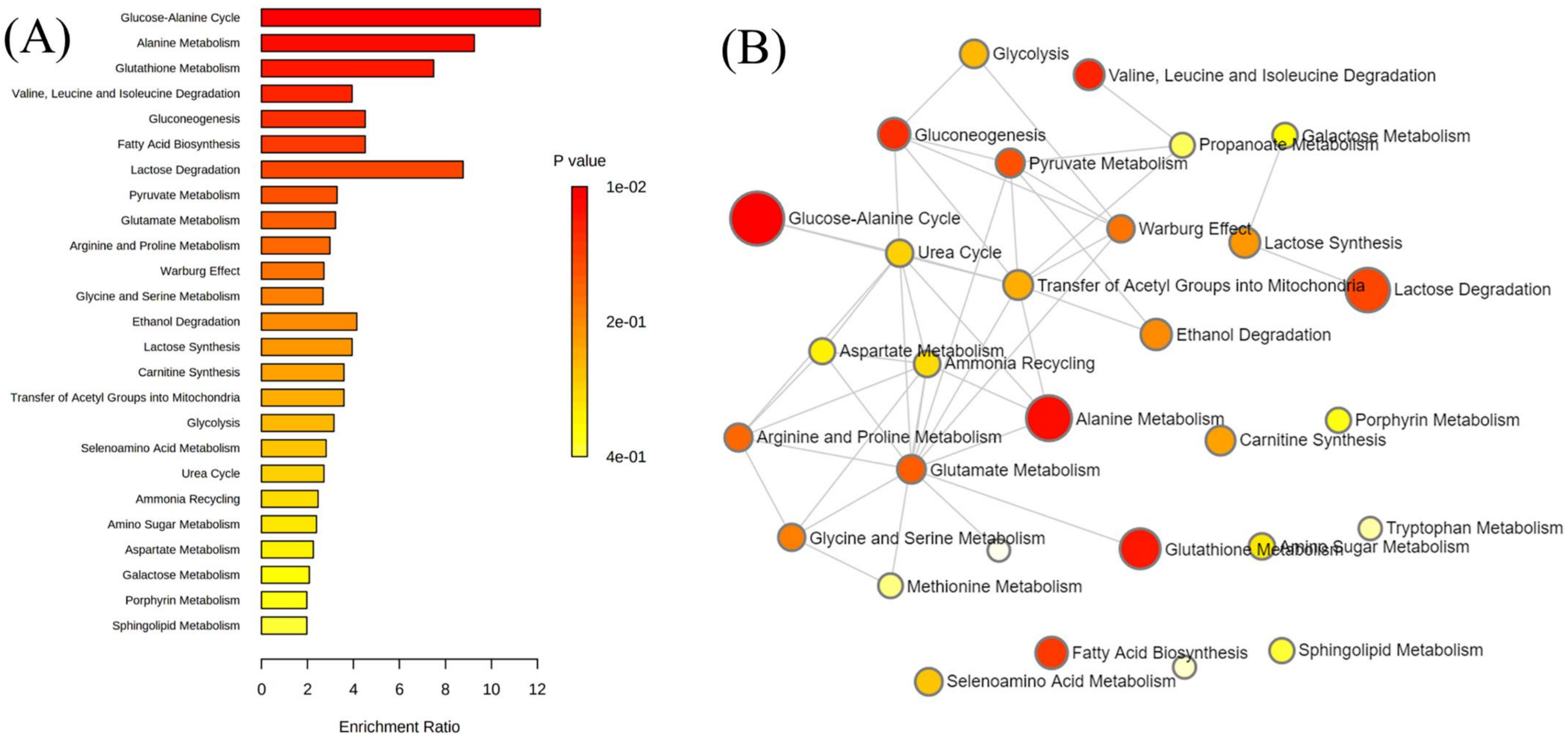

Figure S5. Enrichment analysis of differential endogenous metabolites between AB treated group and model group.

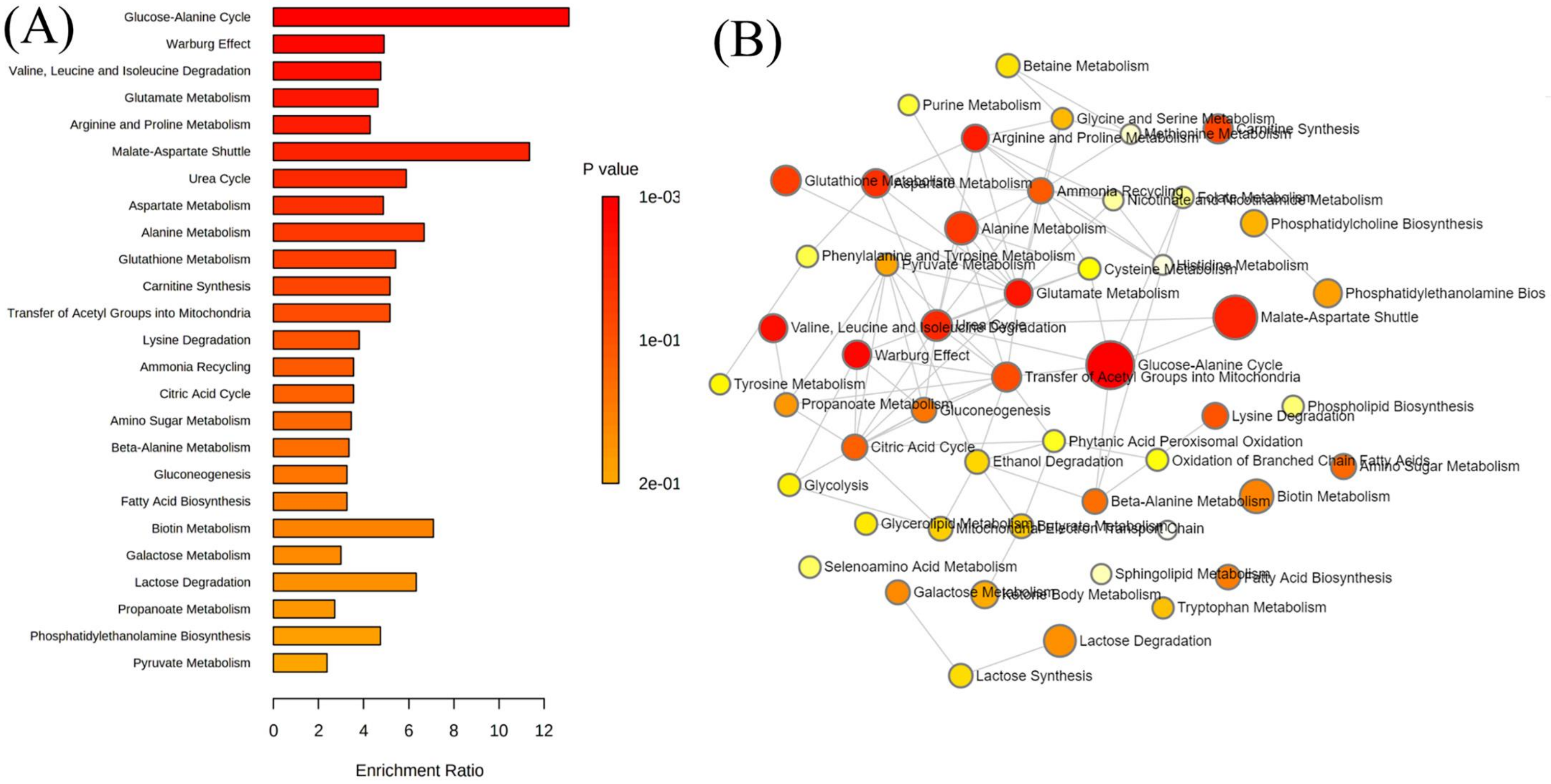

Figure S6. Enrichment analysis of differential endogenous metabolites between sAB treated group and model group.

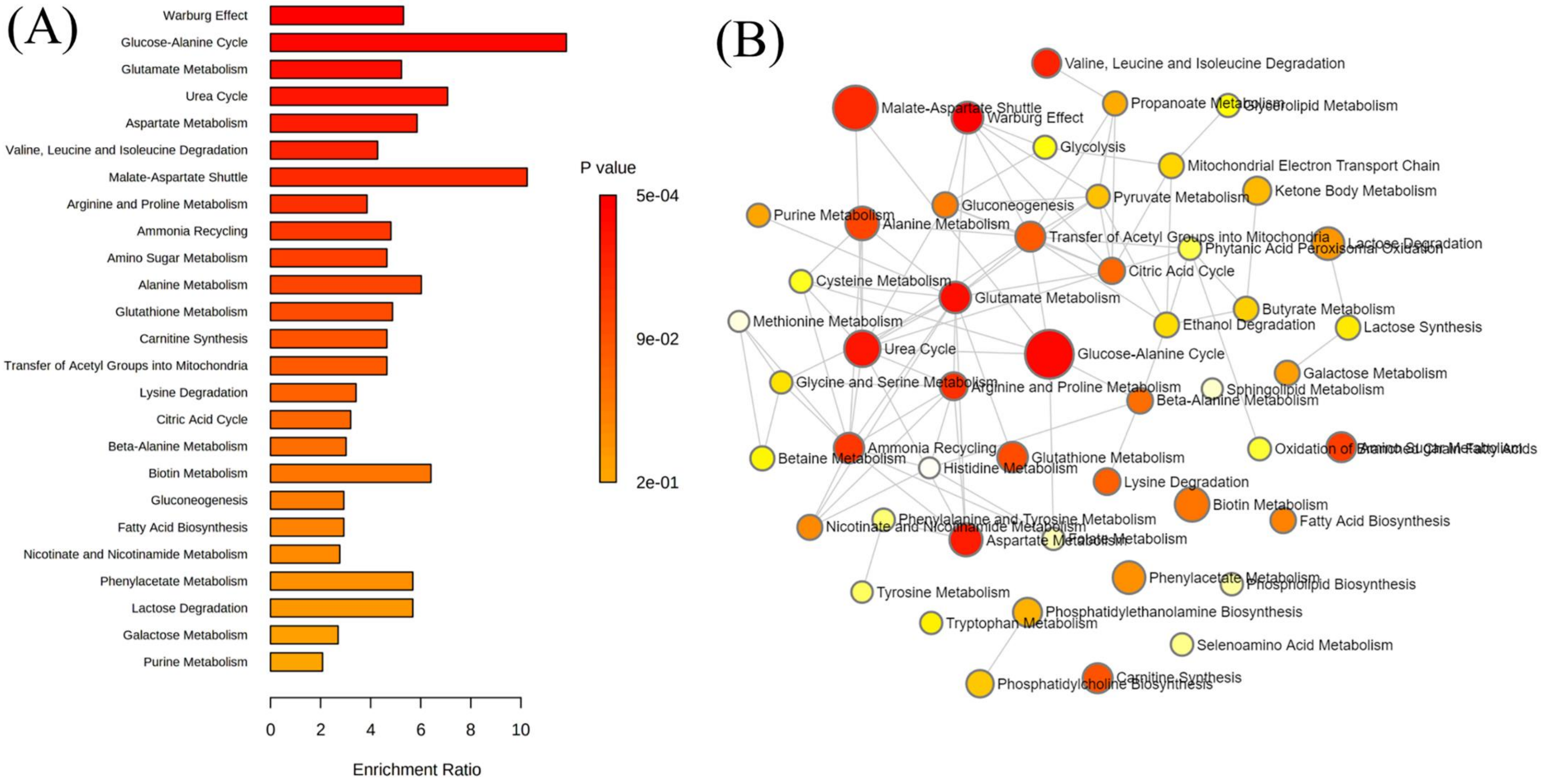

Figure S7. Enrichment analysis of differential endogenous metabolites between ABP treated group and model group.

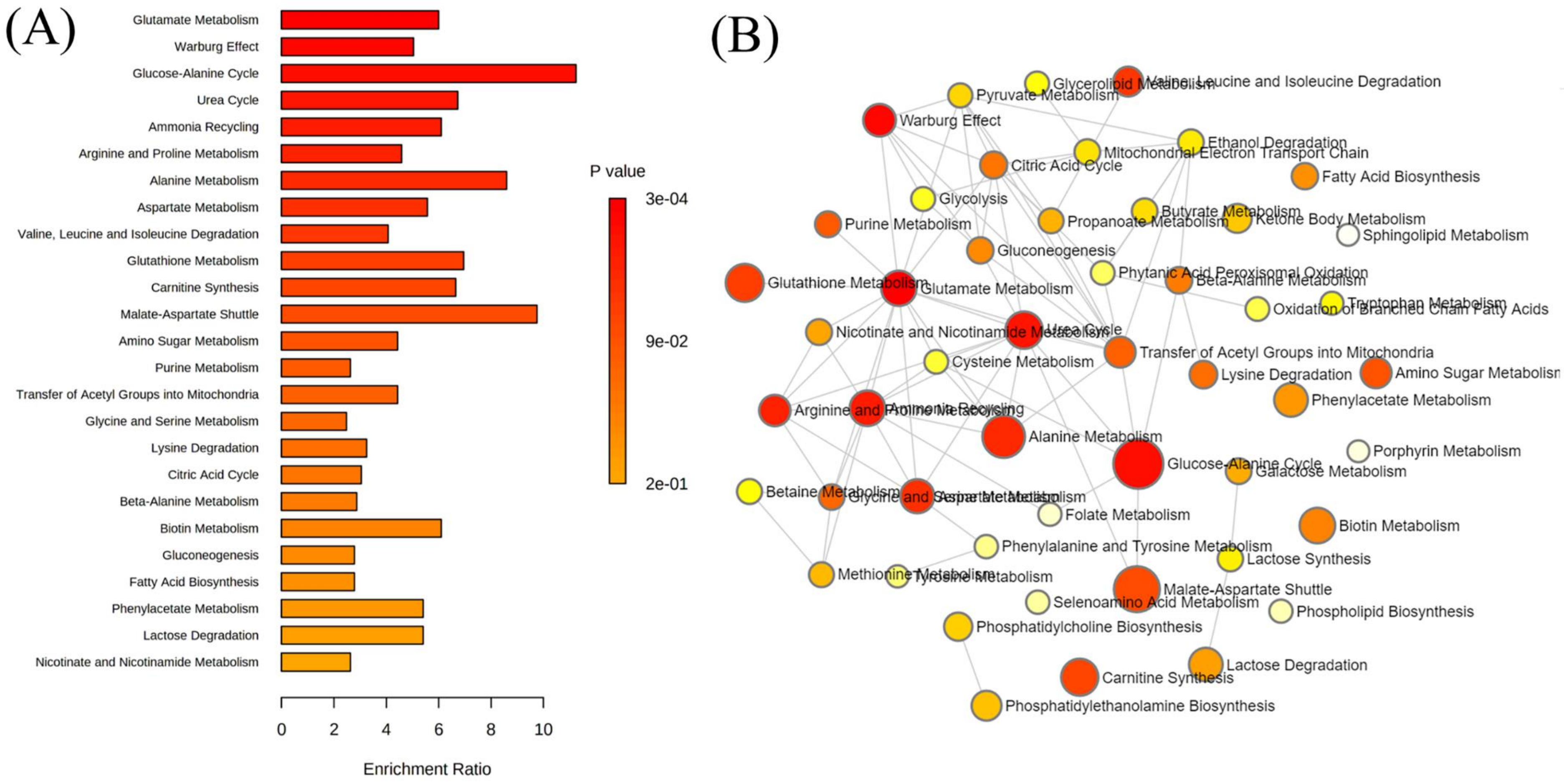

Figure S8. Enrichment analysis of differential endogenous metabolites between sABP treated group and model group
